# Comparision of a Glove-Based Dressing Regimen with Conventional Dressings in Treatment of Superficial Partial-Thickness and Small Deep-Dermal Hand Burns

**DOI:** 10.1055/s-0045-1802330

**Published:** 2025-02-24

**Authors:** Chandrashekhar Subhash Chalwade, Mudunuri Ravi Teja, Raghav Mago, Abhijeet D. Sawant

**Affiliations:** 1 Department of Plastic and Reconstructive Surgery, Seth G. S. Medical College and KEM. Hospital, Mumbai, Maharashtra, India; 2 Department of Plastic Surgery, Topiwala National Medical College and B. Y. L. Nair Hospital, Mumbai, Maharashtra, India

**Keywords:** burn injuries, glove dressing, hand, digit mobility

## Abstract

**Background:**

In cases of burns affecting the hand, minimizing morbidity and disability is crucial. Early mobilization is essential to prevent persistent contractures and optimize functional outcomes by gradually improving range of motion. The purpose of this study was to determine the efficacy of a glove-based dressing regimen in treating superficial partial-thickness and small deep-dermal hand burns compared to conventional dressings.

**Materials and Methods:**

This study was conducted in a burn department affiliated with a tertiary care center over a period of 1 year between January 2018 and December 2018. A total of 75 digits (23 hands of 14 patients) were included in the study with 38 under a case group and 37 under a control group. The primary outcome of study was evaluated by assessing the edema, healing time, and mobility of the affected digit.

**Results:**

Mean healing time in the case group was 12.4 days (range: 11–14, standard deviation [SD]: 0.992). The mean VAS (visual analog scale) score was 8.42 (range: 13–17, SD: 1.136). The percentage increase in mobility was 70.81% in the case group (glove dressing) as opposed to 46.57% in the control group (conventional dressing) at 10 days post-burn, which was statistically significant.

**Conclusion:**

Sterile glove dressing techniques can be handy and a convenient method for dressing of hand burn injuries.

## Introduction

The frequency of hand burns is partly because of its reflex use and partly because it is generally uncovered. 1 Despite accounting for approximately 6% of the total body surface area, the hands are involved in more than 90% of severe burns. 2 Burn injuries account for a significant amount of morbidity and mortality, especially in India. Burns involving the hand is one of the important factors deciding morbidity and disability. 3 A myriad of factors unique to hand have an impact on long-term outcome. It entails wound care, early wound closure, and limitation of pain like any other site of burns. In addition to the above-mentioned factors, reducing digit edema and early mobilization with eventual increasing range of movement (ROM is quite imperative to avoid long-term contractures and maximize function. These factors make the treatment of hand burns challenging. 1 4

Although superficial partial-thickness burns may be managed with local wound care, expedient tangential excision and grafting of deep partial-thickness and full-thickness burns is warranted. In nonsurgical local wound care, special attention must be given to ensure that the dressings are changed at least once a day and are not too bulky to prevent hand mobilization while the wound heals. 1

We studied the glove dressing in our tertiary burn care center in Mumbai, India, which is a public hospital. The purpose of the study was to determine the efficacy of the glove-based regimen in treating superficial partial-thickness and small full-thickness hand burns compared to conventional dressings. We hypothesized that superficial partial-thickness and small full-thickness hand burns can be successfully treated using a glove gauze regimen that facilitates patient compliance, quicker edema reduction, and better ROM.

## Materials and Methods

The study was conducted in the burns department affiliated to a tertiary care center over a period of 1 year between January 2018 to December 2018. Informed consent was obtained from all patients. The authors assert that all procedures contributing to this work comply with the ethical standards of the relevant national and institutional guidelines on human experimentation and with the Helsinki Declaration of 1975, as revised in 2008.

All patients were examined and assessed by senior faculty at our center. All patients between the age group of 18 and 65 years with first and second degree with major superficial dermal burn involving volar as well as dorsal surfaces of digits were included in the study. Those patients with burns more than 30% Total Burn Surface Area (TBSA), large deep dermal or any full thickness burns, keloid tendencies, existing co-morbidities, and pre-existing hand disorders were excluded in our study. A total of 75 digits (23 hands of 14 patients) were included in the study with 38 digits under the case group and 37 digits under the control group. These patients were assigned to the respective group with simple randomization using random numbers generated on a number generator website. 5

The aim of the study was to determine the efficacy of glove-based dressing in comparison to conventional dressing material. The primary objective was to determine the efficacy of glove dressing in terms of reducing edema and improvement in mobility of digits. The secondary objective was to evaluate the healing period of the hand, which was assessed as the number of days in which all hand wounds epithelized completely. Pain was also measured as a secondary parameter.

**Fig. 1:**
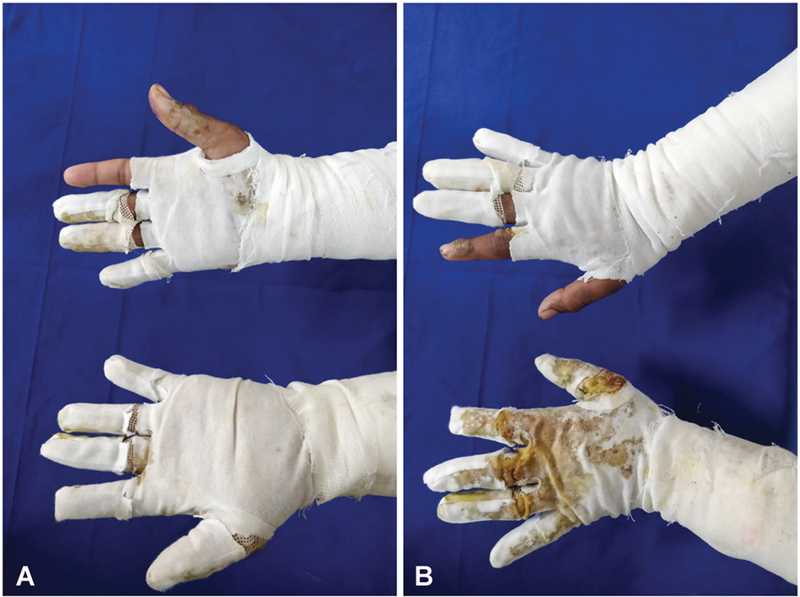
( **A, B** ) Volar and dorsal views of glove-based dressing showing uninvolved digit kept open and each digit dressed separately. In case of soakage or excess exudate, it was overdressed with a larger sized glove.

**Fig. 2:**
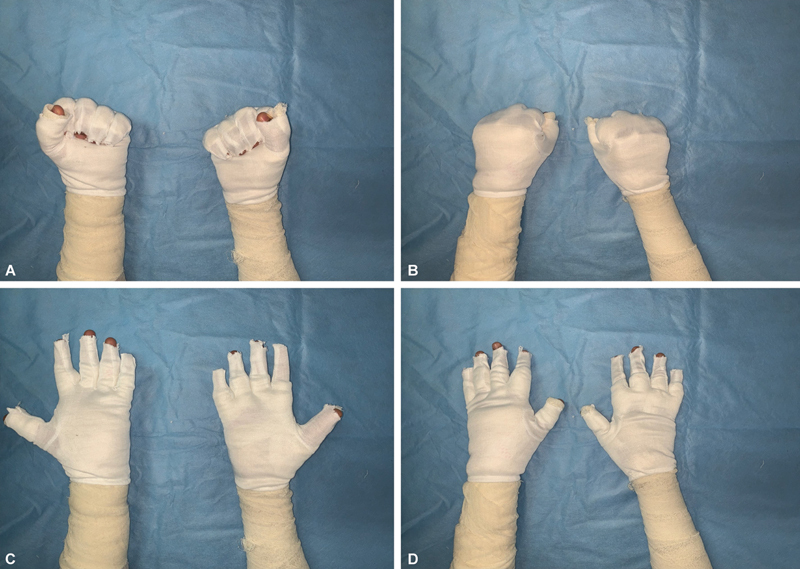
( **A–D** ) Volar and dorsal views of a patient in the study group dressed with glove-based regimen showing ease of digit mobility.

In the case group, 38 burnt digits received standard of care as per our institute's protocol with hand wound dressed in autoclaved stretchable cotton gloves. The hand wound was cleansed with chlorhexidine solution, blisters deroofed, and a thin layer of silver sulfadiazine (SSD) antibiotic cream and nonadherent tulle was applied. This was covered with autoclaved stretchable cotton gloves. The size of the gloves was chosen according to the hand size of patient and extent of hand edema. The thickness of these gloves was 2 mm. If the wound exudate was higher, one large-size glove was put over the first one. For patients who had excessive pain or edema, the finger part of the gloves was cut, and each finger was dressed separately. The tips of the finger portion of the gloves were cut to keep the fingertips open. If any of the fingers were not involved, the respective part of the glove was removed ( Fig. 1 ). In cases where there was exudate or soakage before scheduled dressing, we applied one more glove of larger size over the previous one. At the end of dressing, ease of mobility of digits was confirmed ( Fig. 2 ). On the next dressing change, glove was cut at the sides while removing and was discarded.

**Fig. 3:**
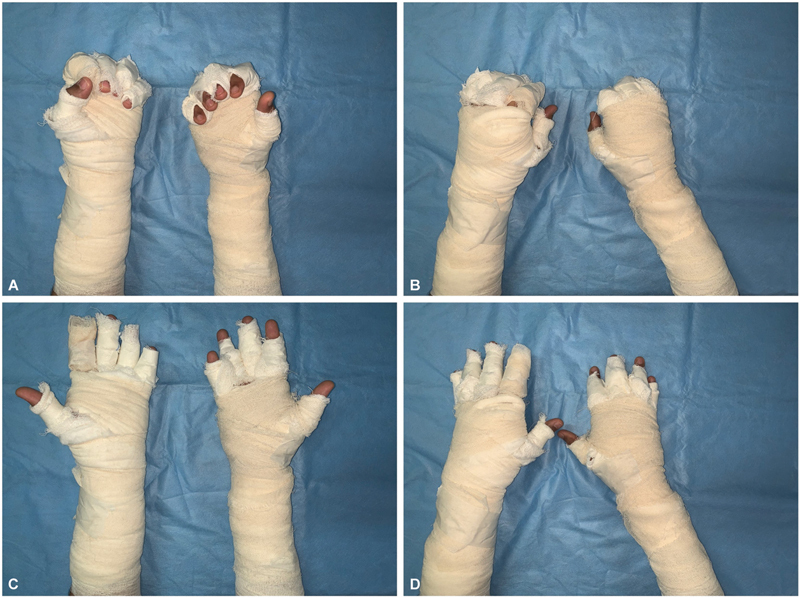
( **a–d** ) Volar and dorsal views of a patient in the control group dressed in conventional manner. The digits were dressed separately to enable similar therapy protocol. Restricted digit mobility can be observed.

In the control group, 37 burnt digits received the same standard of care and wound care with hand dressed with a layer of nonadherent vaseline gauze, an antibiotic cream (SSD), an absorbent gauze layer, and a securing bandage layer. Every digit was dressed separately to enable mobilization ( Fig. 3 ).

Wound cleansing, debridement, and local antibiotic medications were the same in both arms. The cleansing solution used was chlorhexidine scrub solution and water. SSD was the local antibiotic used. Dressings were changed daily. Splinting, therapy protocols, and limb elevation were similar in both the groups. A static functional position within the patient's injury limits was given at night. During daytime, two sessions of supervised mobilization under a therapist and self-performed mobilization at other times were followed.

The primary outcome of the study was determined by assessing the edema and mobility of the affected digits. The edema (E) was measured as circumference in millimeter at the mid-part of proximal phalanx of affected finger on day 0, 5, and 10 (E0 _2,3,4,5,_ E5 _2,3,4,5,_ and E10 _2,3,4,_ corresponding to the second, third, fourth, and fifth digit). These measurements were not taken for the thumb. The readings of multiple digits were averaged to a mean edema measurement for a patient on that day (E0, E5, and E10). The difference between the readings of day 0 and day 10 was noted as the mean edema reduction (dE).

The digit mobility (M) was measured as the distance from the distal tip of the affected digit to the proximal palmar crease in millimeter on day 0, 5, and 10. It was taken as an indirect measure of mobility for the digit (M0 _2,3,4,5,_ M5 _2,3,4,5,_ and M10 _2,3,4_ , corresponding to the second, third, fourth, and fifth digit). This was not recorded for the thumb. It was averaged to a mean digit mobility for a patient on that day (M0, M5, and M10). The difference between the readings of day 0 and day 10 was noted as the mean increase in mobility (dM).

The healing time was noted as the number of days in which the hand burn epithelized completely. Mean for cases and control group was calculated.

The pain was assessed as VAS (visual analogue scale) scores from 1 to 10. It was noted on day 0, 5, and 10 at four times a day (before and after dressing and before and after evening therapy session). These readings were averaged to VAS scores for a patient on that day (VAS 0, 5, 10). The average VAS of patients in the group on day 0, 5, and 10 was calculated as mean VAS scores (VAS 0, 5, 10). The difference in the mean VAS score on day 0 and day 10 was calculated as dVAS cases and control.

Mean, standard deviation, and range for all parameters were calculated using Microsoft Office Excel 2021 program. “ *p* ”-Value (unpaired “ *t* ”-test) was calculated using free an openepi biostatistics calculator available online. 6

## Results

Of the 14 patients on whom the study was conducted, 8 were male and 6 were female patients. In addition, 12 patients sustained burn injuries due to flame burns and 2 patients suffered scald burns. A total of 23 hands burnt were evaluated, of which 6 sustained burns to the dominant hand, 5 sustained burns to the nondominant hand, and 6 to both the hands.

**Fig. 4:**
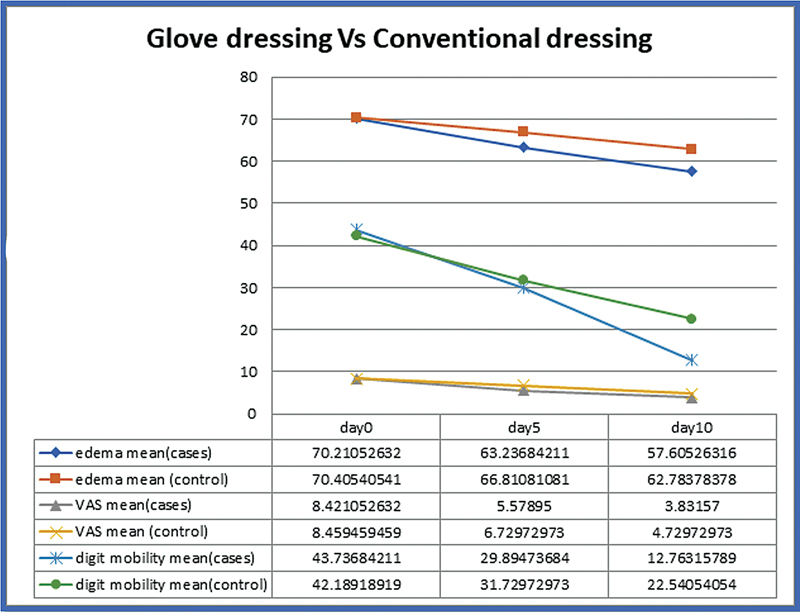
Chart depicting mean digit edema, mean VAS score, and mean digit mobility on day 0, 5, and 10.

The compiled data of mean digit edema, mobility, pain level, and healing time for both the groups are tabulated in Table 1 and a trend is depicted in Fig. 4 .

**Table 1:** Compiled data for mean digit edema, mobility, pain level, and healing time for both the groups (E0, E5, and E10 refer to mean digit edema on day 0, 5, and 10, respectively

Column	Min	Max	Mean	SD	*p* -Value
**E0 cases (** ***n*** ** = 38)**	57	82	70.2105	6.22184	
**E0 control (** ***n*** ** = 37)**	58	80	70.4054	5.77571	
**E5 cases (** ***n*** ** = 38)**	51	75	63.2368	6.06021	**0.009**
**E5 control (** ***n*** ** = 37)**	52	76	66.8108	5.63145	
**E10 cases (** ***n*** ** = 38)**	45	70	57.6053	6.23651	**0.0002**
**E10 control (** ***n*** ** = 37)**	49	72	62.7838	5.52839	
**dE cases (E0–E10)**	11	14	12.6053	0.78978	**0.0000001**
**dE control (E0–E10)**	5	10	7.62162	1.49724	
**VAS 0 cases (** ***n*** ** = 38)**	8	9	8.42105	0.50036	
**VAS 0 control (** ***n*** ** = 37)**	8	9	8.45946	0.50523	
**VAS 5 cases (** ***n*** ** = 38)**	5	7	5.57895	0.55173	**0.0000001**
**VAS 5 control (** ***n*** ** = 37)**	5	8	6.72973	0.59915	
**VAS 10 cases (** ***n*** ** = 38)**	3	6	3.83158	0.71361	**0.0000014**
**VAS 10 control (** ***n*** ** = 37)**	3	6	4.72973	0.76915	
**dVAS cases (VAS 0–VAS 10)**	2	5	2.68947	0.84335	**0.0000014**
**dVAS control (VAS 0–VAS 10)**	2	6	3.72973	0.87078	
**M0 cases (** ***n*** ** = 38)**	36	52	43.7368	4.50036	
**M0 control (** ***n*** ** = 37)**	36	50	42.1892	3.90041	
**M5 cases (** ***n*** ** = 38)**	22	38	29.8947	5.11907	**0.08**
**M5 control (** ***n*** ** = 37)**	26	39	31.7297	3.74647	
**M10 cases (** ***n*** ** = 38)**	8	18	12.7632	2.54085	**0.0000001**
**M10 control (** ***n*** ** = 37)**	15	31	22.5405	4.51291	
**dM cases (M0–M10)**	27	36	30.9737	2.45486	**0.0000001**
**dM control (M0–M10)**	18	22	19.6486	1.15989	
**Healing time, No. of days** **Cases (** ***n*** ** = 38)**	11	14	12.4	0.992	**0.0000001**
**Healing time, No. of days** **Control (** ***n*** ** = 37)**	13	17	15.1	1.136	

Note: VAS 0, VAS 5, and VAS 10 refer to mean VAS scores on day 0, 5, and 10, respectively. M0, M5, and M10 refer to mean digit mobility on day 0, 5, and 10, respectively. dE, dVAS, and dM refer to differences in mean digit edema, mean VAS score, and mean digit mobility, respectively.

The digit edema and mobility improved significantly from day 3 onwards in both the groups. It was better in the study group than the control group on day 5 (nonstatistically significant) and day 10 (statistically significant). The mean healing period in the study group was 12.4 days in contrast to 15.1 days in the control group, with a *p* -value of “0.0000001.” The patients in the study group had significantly less pain compared to those in the control group ( *p* -value 0.0000001 on day 5 and 0.0000014 for day 10; Table 1 and Fig. 4 ).

## Discussion



**Video 1** Video showing comparison of ease of motion of burnt digits with glove dressings (study group) and conventional hand dressings (control group). It can be observed that with conventional hand dressings there is restriction of movements due to bulk of the dressing, whereas patients with glove dressings were able to achieve good and unrestricted hand mobility.


The primary goals in the treatment of hand burns are to prevent anatomic deformities and loss of function. 7 Movement is the key to this as it reduces tendon adherence and capsular contractures. Early initiation of hand therapy improves the final outcome of hand burns. 8 Unfortunately, conventional dressings applied to the burnt hand and individual fingers are often bulky, which limits the mobilization, making them less amenable to effective hand therapy. The application of these dressings is also time-consuming and cumbersome. In addition, the dressings often slip resulting in sub-standard coverage of the burnt areas. 7

The use of sterile glove dressings for management of hand burns is not new. The use of glove therapy was first reported in 1977. 9 The glove, while being inexpensive, provides a perfectly contoured dressing. Furthermore, it is the only dressing modality that freely allows independent movement of individual digits. 10

Although there are various commercial glove dressing materials available in the market, like Acticoat glove, Biobrane glove, Latex gloves, etc., we chose to use simple stretchable cotton gloves for our study, considering easy availability and humid climatic conditions.

**Fig. 5:**
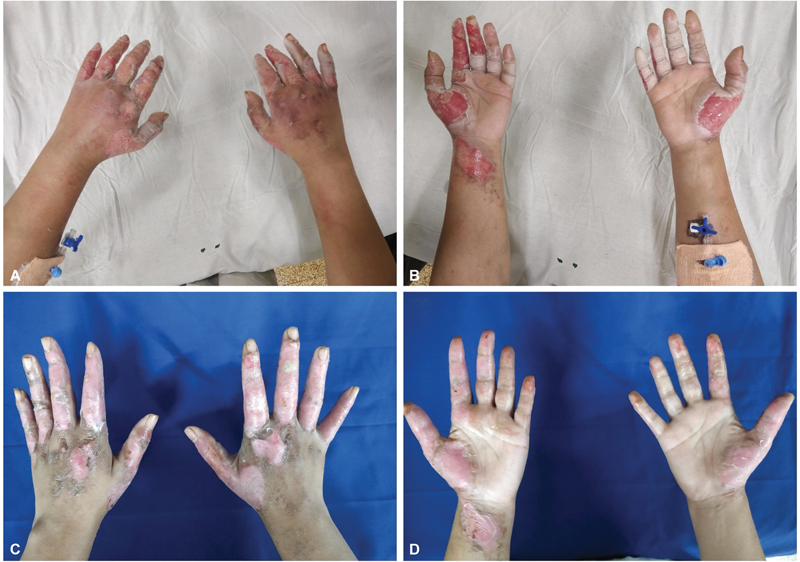
( **A, B** ) Volar and dorsal views of a patient in the study group dressed with glove-based regimen on presentation. ( **C, D** ) Volar and dorsal views of a patient in the study group dressed with glove-based regimen on day 12 showing epithelialized wounds.

We also observed that these glove dressings were less bulky as opposed to the conventional glove dressings. This majorly helped in faster initiation of finger and hand movement. ( Fig. 5 and Video 1 ). Splintage was easier to apply with glove dressing due to its mere compactness post-complete dressing. In a similar study conducted by Vyrva et al where they included burns of all thickness, they observed that 96% of their patients regained full range of motion at the time of final follow-up. The mean time for epithelization was 3.7 weeks in the above-mentioned study. 11 In a study conducted by Busche et al, where Biobrane gloves were used, the mean healing time was 9 days. 12

In our study, we took the tip to palm distance as a parameter to measure the improvement in mobility of fingers. We observed that the percentage improvement in digit mobility was 70.81% in the case group (glove dressing) as opposed to 46.57% in the control group (conventional dressing) at 10 days, which was statistically significant. We observed that digit mobility was closely related to digit edema independent of depth of burn injury. The digit edema started reducing around day 3 and was significantly reduced by day 10 in the case group compared to the control group. The mean time of epithelization was 12.4 days in the glove dressing group and 15.1 days in the conventional dressing group.

**Fig. 6:**
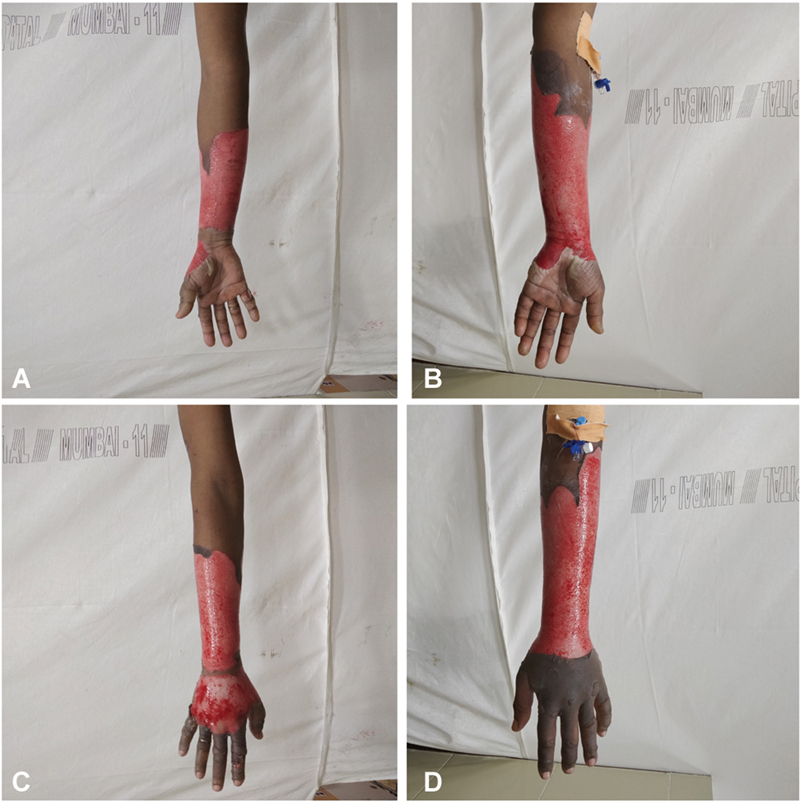
( **A–D** ) Volar and dorsal views of a patient in the control group dressed in conventional manner on presentation.

**Fig. 7:**
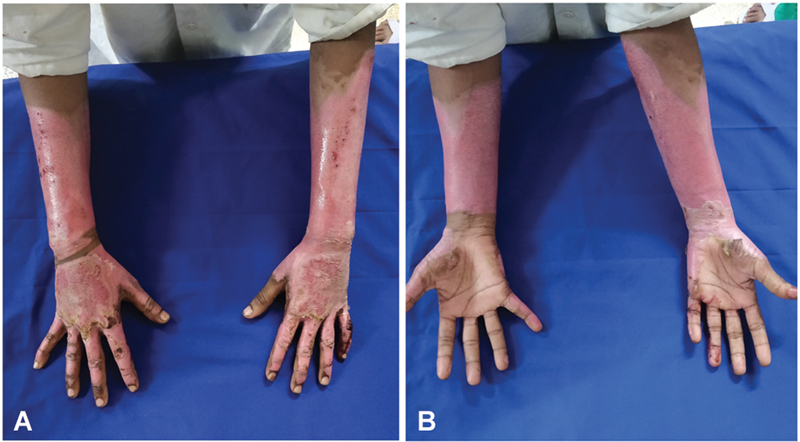
( **A, B** ) Volar and dorsal views of a patient in the control group dressed in conventional manner on day 15 showing partially epithelialized wounds.

We observed that when the patients were given conventional dressings ( Figs. 6 and 7 ), the incidence of contamination and soakage was much higher. The wound culture reports were unremarkable. It was probably related to edema than infection. This would eventually lead to abnormal wound healing with increased fibrosis and unfavorable sequelae like contractures in the long run. 1 These issues were ameliorated with the glove dressing technique.

Another advantage of glove dressing over the conventional one was that the glove dressing was less time consuming, which made the patients more compliant with the whole process of change of dressing. The pain at rest was comparable in both the groups; however, we observed that patients with glove dressings had relatively pain-free hand mobilization when compared to conventional dressing.

As most of our patients belonged to the migrant population from distant areas, follow-up time was of short duration. So, many long-term parameters like scar quality and long-term functional outcomes could not be evaluated. Small sample size is another limitation of our study. Cost benefit due to cotton gloves, which we feel is a major benefit to our patients, could not be analyzed because most of the consumables were from hospital supply and performing cost benefit analysis was not possible.

## Conclusion

The sterile glove-based dressing technique can be handy and a convenient method for dressing of hand burns. Ease of application, ubiquitous availability, patient compliance, and earlier mobilization of hand can greatly aid the tricky management of hand burn injury.
